# Breast and cervical cancer screening in Great Britain: Dynamic interrelated processes

**DOI:** 10.1186/s13561-015-0065-3

**Published:** 2015-10-20

**Authors:** Alexander Labeit, Frank Peinemann

**Affiliations:** 1 School of Health and Related Sciences, Regent Court, University of Sheffield, 30 Regent Street, Sheffield, S1 4DA UK; 2 FOM University of Applied Science for Economics & Management, Leimkugelstr. 6, 45141 Essen, Germany

**Keywords:** Dynamic panel probit model, Bivariate probit, Preventive medicine, State dependence, Screening, Health check-ups, C33, C51, I19

## Abstract

No previous analysis has investigated the determinants of screening uptake for breast and cervical cancer screening for possible spillover effects from one type of screening examination to the other type of screening examination with a dynamic bivariate panel probit model. For our analysis, we used a dynamic random effects bivariate panel probit model with initial conditions (Wooldridge-type estimator) and dependent variables were the participation of breast and cervical cancer screening in the recent year. The balanced panel sample consisted of 844 women from the British Household Panel Survey (BHPS) from the time period 1992 to 2008. Our analysis showed the high relevance of past screening behaviour and the importance of state dependency for the same and the other type of cancer screening examinations even after controlling for covariates and unobserved heterogeneity. The uptake for breast and cervical cancer screening was higher when the same screening examination was done one or three years earlier. This result is in accordance with the medical screening programmes in Great Britain. With regard to breast and cervical cancer screening positive spillover effects existed between screening examinations in the third order lags. Women with a previous visit to a general practitioner and individuals in the recommended age groups had a higher uptake for breast and cervical cancer screening. Other socioeconomic and health related variables had non-uniform results in both screening examinations. Promoting the uptake of one female prevention activity could also enhance the uptake of the other prevention activity.

## Background

Individuals can attend different health check-ups in the National Health Service (NHS). These include breast cancer screening, cervical cancer screening, blood pressure check, cholesterol test, dental screening and eyesight test. The NHS Breast Screening Programme has established policy rules on the invitation to a mammography and the NHS Cervical Screening Programme has established policy rules for the invitation to a Pap smear test. High participation rates are an important aim for both screening examinations, because mammographies and cervical smear tests give the possibility of early detection of breast cancer and prevention of cervical cancer. Breast and cervical cancers have a high chance to be cured if detected at an early stage [1, 2].

Previous research with the BHPS has analysed the uptake for breast and cervical cancer screening in several studies [3, 4], however the uptake was typically only analysed for one type of screening examination: Labeit et al. (2013) analysed the uptake of cervical cancer screening until 2008 [5] and Carney et al. (2013) analysed the uptake of breast cancer screening until 2008 [6] and Sabates et al. (2008) estimated breast and cervical cancer screening not as separate equations and potential correlated processes [3]. None of these empirical analyses investigated the uptake of breast and cervical cancer screening examinations simultaneously and if there are possible spillover effects from one type of examination to the other type of examination with a dynamic bivariate panel probit model.

First, the regulations for the breast and cervical cancer screening are described: the policy rules for the invitation with the age of invitation, time periods between different screening examinations and the different invitation rules for England, Scotland and Wales and the location in which the screening examination is typically done. Second, economic models and the relevance for prevention activities and potential weaknesses are discussed and extensions of these models with non-economic factors are introduced. Also, empirical evidence for own analysed variables is discussed and third, the used dataset is described.

For breast and cervical cancer screening examinations, explicit policy rules exist detailing the frequency of screening examinations depending on age limits, cancer history of first-degree relatives and screening examinations in previous years. The NHS Breast Screening Programme and the NHS Cervical Screening Programme are both public health programmes in Great Britain and they are population based screening programmes^1^. The access to both screening programmes is not possible outside the relevant age groups and time intervals. Both programmes issue invitations on regular schedules that cannot be altered and the women will then make the appointment themselves. Outside of these programmes mammography or smear test could take place in order to investigate a symptom or in a local surveillance arrangements.

The NHS Breast Screening Programme (NHSBSP) is a national prevention programme in Great Britain which offers women a mammography [7]. This programme is an early detection programme for an invasive disease and in some cases it detects breast cancer in situ. The incidence of breast cancer rises around age 50 and about 80 % of new cases were diagnosed in women aged 50 and over, and 14% of all new cases were detected in the age group between 60 and 64 [8]. Relevant risk factors are reproductive and hormonal factors, obesity, alcohol and physical activity [9]. A proportion of about one quarter of breast cancer cases can be linked to largely modifiable such as lifestyle and environmental factors [10].

Originally, only women between age 50 and 64 were invited for the first screening and every three years thereafter, but since 2003 women between age 65 and 70 years have been invited. More frequent examinations are now recommended for high risk women (e.g. a first-degree relative (mother or sister) with breast cancer at a young age). Mammographies are typically conducted in acute hospital trusts or in mobile van units.

The NHS Cervical Screening Programme (NHSCSP) is a national prevention programme in Great Britain which offers women a smear test at different time intervals depending on age [11]. The aim of this programme is to prevent cancer by detecting and treating abnormalities in an early stage which, if left could develop into cancer. One of the most important risks factors for cervical cancer is infection with the human papilloma virus (HPV). HPV is an infection that is spread through sexual contact, and as a consequence, about half of all women will be infected during their whole lifetime [12]. Some of the women with an HPV infection will develop cervical cancer. Younger women are more susceptible to develop cervical cancer and younger females who have sexual intercourse at an early age and with a higher number of sexual partners will have a higher chance to develop cervical cancer in comparison to older women with few sexual partners [13]. In 2008, as part of the NHS childhood vaccination programme, the HPV vaccination was introduced routinely to all girls aged 12 to 13 in England. Other risk factors for developing cervical cancer are an increased duration of oral contraceptive use and a young age at first full term pregnancy [14]. The registration of newly diagnosed cases of cervical cancer start to rise in the age group 20 to 24 and it is highest for women between 25 and 49 and has one peak for women aged 25 to 29 and another peak for women aged 45 to 49. Therefore, incidence and mortality rates have different age distributions for both female cancer types with the consequence of different ages for the first and last invitation by these programmes.

The age for the first invitation and last invitation to cervical cancer screening varies by region in Great Britain: age 25 in England since 2003, or 20 in Scotland and Wales, and 20 in England before 2003 [15, 16]. Between the age of the first invitation and 49, there is a 3 yearly recall period in all parts of Great Britain since 2003, although before 2003 there was a 3 to 5 yearly recall period policy depending on the Primary Care Trust, with the majority of Primary Care Trusts following a 3-year policy [17]. The policy of a uniform 3 year recall period for women between ages 25–49 was implemented in England in 2003 after a recommendation by the Advisory Committee on Cervical Screening following an audit funded by Cancer Research UK and at the same time women between 20 and 24 were withdrawn from the programme in England. It was concluded that a 3-year recall policy appeared most effective after an analysis of UK data [18]. No information was available for us on how quickly each Primary Care Trust in England implemented the changes to the recall policy. Cervical cancer screening is offered to women aged 50 and over every three years until age 60; every three years until age 64 in Wales; and every five years in England until age 64 [15]. Before 2003, a majority of women were screened every three years [17]. Women who reach the age of 60 in Scotland, and age 65 in England and Wales are excluded from the recall system and no longer invited for cervical cancer screening unless they have had a recent abnormal result in one of the previous three tests. Cervical cancer screening examinations can be done by a General practitioner (GP) or appropriate service provider such as a woman or family clinic or the genito-urinary medicine (GUM) department of a hospital [11]. These examinations are offered as an ‘additional service’ by a GP and most cervical screening examinations are undertaken by a female practice nurse. Liquid based cytology (LBC) which is an improved way of preparing cervical samples for examination in the laboratory was introduced between 2003 and 2008 [19] and the conversion to LBC was completed in October 2008 [11]. For breast and cervical cancer screening, periodic invitations are sent out routinely to women by the programmes which are based on the NHS registers of GP patients.

The rules of these programmes are relevant for analysing the uptake of both screening examinations, because according to these policy rules women are invited at certain time intervals for the screening examinations. There is an increased likelihood of participating in a screening examination after the recommended time interval. Also, for both screening examinations, an inconclusive test in the actual year may make it necessary for a follow-up control screening examination in the next year. Control screening examinations as a follow-up are typically done for breast and cervical cancer screening to check unclear test results.

### Economic models and existing empirical evidence

Economic models for the demand of health care in general and for preventative services in particular are based on human capital models [20]. This theoretic framework has been used for modelling the demand of primary and secondary prevention [21]. Therefore, it is also used in the case of female cancer screenings such as mammography and smear testing [22]. Cancer screening examinations have a self-protection function and improve early detection of these cancer types and health outcomes [23]. The problem with economic models for the demand of health care, including prevention care, is that two important aspects are typically not considered at the same time in detail: the distinction between acute and preventative care, and uncertainty. Some dynamic economic models for the demand of health care take only uncertainty into consideration. However, there is no distinction made between acute and preventative care [24]. Acute care describes the consumption aspect of health, whereas preventative care describes the investment aspect. The Grossman model makes the distinction between acute and preventative care, but uncertainty is not considered in this model [25]. There is only one economic model which explicitly takes the demand for preventative health care and uncertainty in a stochastic dynamic framework into consideration [26]. It is also necessary to analyse non-economic factors for the uptake of screening examinations and many studies neglect non-economic factors [27]. The conceptual framework of our analysis is based on a human capital approach and inclusion of further non-economic factors.

Hypotheses can be generated for the effect on the demand for preventative services concerning age, education and household income. It is known that age can have different effects on the demand for breast and cervical cancer screening [22, 28]. With respect to breast and cervical cancer screening, policy rules for issuing the invitation do exist that state explicitly the time interval for the screening examinations. For the recommended age intervals, uptake should be higher than for non-recommended age intervals. On one hand, according to the Grossman model, health depreciates as the age increases, and the need to maintain health increases. As a consequence, the demand for prevention activities, such as breast and cervical cancer screening would increase with age. On the other hand, older women have a shorter life span and pay-off period for their investment in breast and cervical cancer screening. Therefore, the effect of increasing age on the uptake for both screening examinations cannot be predicted with confidence. Empirical studies often find a negative relationship between age and uptake for breast and cervical cancer screening [22, 29]. A higher educational level may be expected to lead to an increase in the uptake for breast and cervical cancer screening, because women with a higher education level have a higher efficiency of health production, self-efficacy, motivation, awareness and knowledge about the importance of prevention [21, 30]. However, there is also contradicting empirical evidence; for example, it had been found in one empirical study from Denmark that a high level of education increased the relative risk for never taking part in a mammography in comparison to always taking part [31]. A higher household income leads to an increase of time in perfect health and therefore the demand for both screening examinations should increase [20]. The effect of increasing household income on breast and cervical cancer screening uptake was confirmed in several studies [22, 28, 32]. It can be expected that the effect of increasing household income should be either weaker or not existent in Great Britain when compared to other countries, because both cancer screening examinations are free of charge in Great Britain.

The chance that a woman will visit a breast and cervical cancer screening examination is dependent on further non-economic factors such as previous screening history and individual and household characteristics. We generate hypotheses and discuss existing empirical evidence. The history of breast or cervical cancer screening examinations has a predictive value for uptake in the recent period, i.e. the past screening behaviour is correlated with the current behaviour [6, 27, 30]. Cohabitation status can be an indicator of social support, women living in a partnership being able to exchange information with their partners about health check-ups in general and cancer screenings in particular. This hypothesis was confirmed by two empirical studies which analysed breast and cervical screening examinations. It has been found that women who lived in cohabitation in Sweden had a higher uptake for both screening examinations [33, 34]. In Great Britain, two studies included the number of children as a control variable and found that women with a higher number of children attended breast and cancer screening examinations less often [30, 35]. Employment was added as a further control variable, because women who work may have higher opportunity costs in comparison to unemployed and retired women. In a systematic review that analysed the influence of different determinants on the uptake of different health check-ups, it was found that the influence of employment for the uptake of breast and cervical cancer screening was mixed [27]. The GP plays a role as gatekeeper in the health care system and can give advice and information about the importance of breast and cervical cancer screening examinations. Thus, the uptake of both health check-ups should be enhanced by previous GP visits [36, 37]. Registration with a GP is a necessary condition for receiving an invitation letter for breast and cervical cancer screening and routine periodic invitations are sent from the National Health Service (NHS) registers of GP patients according to the recommended interval for breast and cervical cancer screening. Change of residence to a new address of a woman lowers the chance of receiving an invitation letter. A lower uptake for cervical cancer screening was found for women in Great Britain who had changed residence and address in one study [35], however not in another study [30]. Poor self-perceived general health status could increase participation in screening examinations to find the reason for the poor health status and to invest in better health. This appears to be the case for general health check-ups such as blood pressure check and cholesterol test, but not for female specific cancer screenings such as the mammography and the smear test [28]. Psychological factors such as fear and anxiety of having a cancer diagnosis confirmed in these female specific health check-ups may prevent women from attending a female cancer specific health check-up and these psychological factors are related to a poor health status [38]. Furthermore, women with poor health status may be unable to visit the screening location such as the GP, family clinic or mammography unit, because of physical limitations. For women with poor health decreased uptake of mammograms and pap smears has been reported in the empirical literature. There are mixed results for the effect of poor health status or comorbidities: in one study screening rates decreased as comorbidity increased measured by an index of comorbidity, which was adapted from the Charlson comorbidity index [39], however in two other studies mammography utilization was higher among women with 3 or more stable comorbidities than among those without comorbidities [40, 41]. Smoking can serve as an indicator for the weakened preference for health in comparison to other goods and smoking individuals show risk taking behaviour [42]. Women who smoke have poorer preventative health habits such as a reduced level of physical activity in comparison to non-smoking women [43]. The predicted influence of smoking was empirically confirmed for breast cancer screening with a lower uptake for smoking women than for non-smoking women [22]. For some women with non-white ethnic origin, cultural barriers could exist for both screening examinations, because breast and cervical cancer screening can be experienced as invading medical procedures into the private sphere of a woman. In an empirical investigation, ethnicity was the most important predictor for cervical cancer screening, with white British women having a higher uptake than women of other ethnicity [44]. One further study demonstrated a positive association between the uptake of breast cancer screening and other preventative health check-ups [45]. A systematic review indicated a positive influence of taking part in screening examinations and future health-promoting behaviours and a majority of the studies showed a positive association between both screening examinations [46]. However, all of these studies were cross-sectional or cohort studies and covered a short time span, and studies did not analyse the temporal sequence of screening examinations over a longer period with panel data.

### Data

The BHPS was used for the analysis of breast and cervical cancer screening, the BHPS being an annual survey of households in the UK. It involves a national representative sample of more than 5,000 households and with individuals age 16 and over [47]. The survey began in 1991 and all the original individuals were interviewed annually unless they dropped out. In our analysis and construction of the balanced sample, only individuals from England, Scotland and Wales were selected, because data collection did not start in Northern Ireland until wave 11. For the construction of the balanced panel, 17 years of information were used: from 1992 to 2008, because in the first wave only a few individuals were interviewed in 1991, most in 1992.

Questions about participating in breast and cervical cancer screening were in every wave from the start of the panel survey in 1991 until 2008. For the analysis of breast and cervical cancer screening behaviour, only women were included in the sample. For women to be included in our analysis, provision of both cancer screening examinations had to be from the NHS; females with private provision or with NHS and private provision for these health check-up have been excluded from the analysis. The dependent variable takes the value of 1 in a specific year if the breast cancer screening or cervical cancer screening was done and 0 if not. For analysing the policy changes for breast cancer screening and cervical cancer screening, dummy coding was chosen: for breast cancer screening and the age group 65–70, all years before and including 2002 were coded with 0 and all the following years with 1; for cervical cancer screening and the age group 25–49, all years before and including 2003 were coded with 0 and all the following years with 1.

## Methods

To model the dynamic nature of screening examinations and because uptake is a binary variable, a dynamic random effects (RE) panel probit model was used to estimate the uptake of both screening examinations over the panel period from 1992 to 2008. In such a model, it is possible to estimate the effect of state dependence from the same type of screening examination and spillover effects from the other type of screening examination and also to control for unobserved heterogeneity and the correlation between the individual specific random effects terms. Possible dynamic spillover effects can exist in a bivariate model from one type of screening examination (e.g. breast cancer screening) to the other type of screening examination (e.g. cervical cancer screening) and vice versa. The influence of household and individual characteristics on uptake can also be analysed within such a model.

The formal presentation extends the dynamic random effects univariate panel probit model to the dynamic random effects bivariate panel probit model and follows Alessie et al. [48] and Devicienti et al. [49]. One possibility for estimating a dynamic random effects panel probit is the Wooldridge estimator which specifies a relationship between the unobserved time-invariant individual effect and the observed characteristics and initial conditions. The univariate case for the Wooldridge estimator can be modelled with the following 3 equations [50].

1 $$ {y}_{it}^{*}=y{\hbox{'}}_{i,t-1}\gamma +x{\hbox{'}}_{it}\beta +{c}_i+{\mu}_{it} $$

2 $$ {c}_i={a}_0+{a}_1{y}_{i1}+{\overline{X}}_i^{\hbox{'}}{a}_2+{\alpha}_i $$

3 $$ {y}_{it}=\left\{\begin{array}{l}1, if\kern0.5em {y}_{it}^{*}>0\\ {}0, otherwise\end{array}\right.\ t=2,\dots, T $$

In the first equation ${y}_{it}^{*}$ indicates the unobserved latent variable of an individual *i* at a given time *t* for taking part in a specific screening examination, *y*_*i,t-1*_ is the screening examination decision of the individual *i* in period *t-1*, *γ* is the coefficient for this variable, *x* is a vector of time variant and time invariant covariates, *β* is the vector of coefficients associated with these covariates, *u*_*it*_ is the random error term of an individual *i* in period *t* with normal distribution with zero means and unit variances. *c*_*i*_ indicates the individual specific random effect which is modelled according to the second equation and ${\overline{X}}_i$ are longitudinal averages of an individual *i* for specified variables. *a*_0_, *a*_1_, *a*_2_ are parameters which have to be estimated and *α*_*i*_ is a term with normal distribution with zero mean and variance ${\sigma}_{\alpha}^2$. A normal density for the individual specific random effect is assumed. Correlation between the individual specific random effect and time-varying variables of an individual is allowed by including the average of these variables over the whole panel observation period in the second equation [51]. Time-varying variables included in the second equation can be partitionated into an actual (transitory) and permanent (averaged) component for the estimation. The third equation gives the observed binary outcome *y*_*it*_ of taking part in one screening examination for an individual *i* in period *t*. Such a model can estimated with standard software.

The estimation of such a dynamic random effects panel probit model can be extended for the bivariate case in the following way:

4 $$ {y}_{1 it}^{*}={\gamma}_{11}{y}_{1i,t-1}+{\gamma}_{12}{y}_{2i,t-1}+x{\hbox{'}}_{it}{\beta}_1+{c}_{1i}+{\mu}_{1 it} $$

5 $$ {y}_{2 it}^{*}={\gamma}_{21}{y}_{2i,t-1}+{\gamma}_{22}{y}_{2i,t-1}+x{\hbox{'}}_{it}{\beta}_2+{c}_{2i}+{\mu}_{2 it} $$

With

6 $$ {y}_{jit}=1+\left[{y}_{jit}^{*}>0\right]j=1,2\kern0.5em t=2,\dots, T $$

${y}_{jit}^{*}$ is the chance of a woman *i* in period *t* to have a cancer screening examination *j* (breast or cervical cancer examination) expressed as latent variable. *β* = (*β*_1_, *β*_2_) is the vector of coefficients associated with the covariates in equation (4) and (5). The assumptions for the error terms *μ*_1*it*_ and *μ*_2*it*_ are a bivariate normal distribution with a zero mean and a unit variance for each error term and independency over time and a cross-equation covariance of *ρ*. Individual specific random effects are *c*_1*i*_ and *c*_2*i*_ for breast and cervical cancer screening with a bivariate normal distribution with variances comorbidity and ${\sigma}_{c2}^2$ and covariances *σ*_*c*1_, *σ*_*c*1_, *ρ*_*c*_.

The equation (6) gave the observed binary outcome for breast cancer screening (*y*_1*it*_) for the individual woman *i* in period *t*, which was equal to 1 if the woman *i* had a breast cancer screening examination in period t and 0 otherwise. Analogue the observed binary outcome for the individual woman *i* in period *t* for cervical cancer screening (*y*_2*it*_) was equal to 1 if the woman *i* had a cervical screening examination in period t and 0 otherwise.

The inclusion of lagged dependent variables for breast and cervical cancer screening makes a distinction between unobserved heterogeneity and state dependence possible, and potential dynamic spillover effects from one type of cancer screening to the other type of cancer screening for the women can be analysed. Additionally, such a specification allows for correlated unobserved heterogeneity between the two processes and takes into account the two initial conditions of the separate processes. It is also possible to analyse if the correlation in the observed outcomes for breast and cervical cancer screening is caused by correlation of unobserved heterogeneity (*ρ*_*c*_ ≠ 0) or by spillover effects between both screening examinations (*γ*_12_ and *γ*_21_ ≠ 0). The dynamic random effects bivariate panel probit model considered in equations 7 and 8 can be simplified in some cases. If the coefficients *γ*_12_ and *γ*_21_ are both 0, both equations could be estimated independently and so both equations could be estimated as a dynamic random effects univariate panel probit model. If the coefficients *γ*_12_ ≠ 0 or *γ*_21_ ≠ 0 and the individual specific random effects and the error terms of the 2 equations are independent, which requires *ρ* = 0 and, *ρ*_*c*_ = 0 then equation 7 or equation 8 can be estimated as separate equations. If this is not the case, then for all other cases both equations have to be estimated jointly for getting consistent estimates.

7 $$ {y}_{1 it}^{*}={\gamma}_{11}{y}_{1i,t-k}+{\gamma}_{12}{y}_{2i,t-k}+x{\hbox{'}}_{it}{\beta}_1+{a}_{10}+{a}_{11}{y}_{1i1}+{a}_{12}{y}_{2i1}+{\overline{X}}_i^{\hbox{'}}{a}_{13}+{\alpha}_{1i}+{u}_{1 it} $$

8 $$ {y}_{2 it}^{*}={\gamma}_{21}{y}_{1i,t-k}+{\gamma}_{22}{y}_{2i,t-k}+x{\hbox{'}}_{it}{\beta}_2+{a}_{20}+{a}_{21}{y}_{1i1}+{a}_{22}{y}_{2i1}+{\overline{X}}_i^{\hbox{'}}{a}_{23}+{\alpha}_{2i}+{u}_{1 it} $$

with

9 $$ {c}_{1i}={a}_{10}+{a}_{11}{y}_{1i1}+{a}_{12}{y}_{2i1}+{\overline{X}}_i^{\hbox{'}}{a}_{13}+{\alpha}_{1i} $$

10 $$ {c}_{2i}={a}_{20}+{a}_{21}{y}_{1i1}+{a}_{22}{y}_{2i1}+{\overline{X}}_i^{\hbox{'}}{a}_{23}+{\alpha}_{2i} $$

An alternative instead of using the Wooldridge estimator would be using the Alessie estimator [48] which extends the Heckman estimator [52] to the bivariate case for the dynamic random effects panel probit. However, advantages of using the Wooldridge estimator in comparison to the Alessie estimator are: the numbers of parameters which have to be estimated are smaller and higher order dynamics with lagged dependent variables can be more easily modelled. A dynamic specification with 1-year, 2-year, 3-year lagged dependent variables as explanatory variables takes into account the existence of policy rules for the invitation exist for breast and cervical cancer screening in Great Britain and also that screening examinations from the previous year could have an inconclusive result with the consequent need for a further screening examination. This was especially relevant for the cervical cancer screening examination before the introduction of LBC. For these reasons, we used the Wooldridge-type estimator and used 3 lags for the modelling of the dynamic structure of both screening processes. For the initial conditions for breast or cervical cancer screening for an individual woman *i* we used the information about breast or cervical cancer screening in the three waves from 1992 to 1994.

The Wooldridge estimator assumes a balanced panel, because selection and attrition can depend on initial conditions. It allows for different initial statuses to have different missing data possibilities. Therefore, it is not explicitly necessary to model selection and attrition as a function of initial conditions [53]. Important assumptions for the estimation of the dynamic random effects univariate and bivariate panel probit model are: the distributional assumptions on the initial conditions are correct and the relationship between the unobserved time-invariant individual effect and the mean of the observed characteristics are correctly specified. A further assumption for an unbiased estimation with regard to initial conditions for breast and cervical cancer screening is the assumption that unobserved breast and cervical cancer screening examinations that happened prior to the panel observation period are uncorrelated with the observed breast and cervical cancer screening examinations and if these assumptions are violated the estimation results could be biased. The breast cancer screening programme (NHSBSP) and cervical cancer screening programme (NHSCSP) were introduced in 1988 before the beginning of the BHPS. For our estimation technique, it is assumed that female cancer screening examinations which had been undertaken before the first wave of the BHPS are uncorrelated with the cancer screening examinations recorded in the BHPS. If this assumption is violated, the inclusion of initial conditions of health check-ups for the years 1992 to 1994 could result in biased estimates for our regressions.

The dynamic random effects univariate and bivariate panel probit models are estimated for breast and cervical cancer screening with lagged dependent variables as explaining variables and lags were used up to order 3. This econometric specification was also chosen in other related studies [5, 6, 30]. In a first step, a dynamic univariate pooled probit with the assumption of exogenous lags was applied and the dynamic random effects univariate panel probit for the breast and cervical cancer screening were estimated. In a second step, a dynamic bivariate pooled probit model and a dynamic random effects bivariate panel probit were estimated^2^. These estimations of the bivariate probit models were compared with the estimations for the univariate probit models and also both bivariate probit models were compared with each other. As a robustness check for the dynamic random effects bivariate panel probit, a further specification was tested for the possible endogeneity of a GP visit for breast and cervical cancer screening. Additionally, unbalanced panels were estimated and their results were compared with balanced panels.

## Results

The balanced panel for breast and cervical cancer screening consisted of 844 women with 11816 observations from 1992 to 2008. Breast and cervical cancer screening information was available over the entire panel period. In our analysis, we categorized age groups for breast cancer screening as follows: 16 to 49 (reference category), 50 to 64, 65 to 70, age 71 and over. For cervical cancer screening, we also categorized by age groups: 16 to 19 (reference category), 20 to 24, 25 to 49, 50 to 64, age 65 and over. Household income was deflated and transformed in per capita income using the modified OECD scale to adjust for household size and needs [54]. Actual income was defined as the total equivalised and deflated household annual income divided by 100 in the actual wave and averaged (permanent) household income was defined as annual household income over the 17 years between 1992 and 2008. The International Standard Classification of Education (ISCED) was used for the categorisation of educational levels with tertiary, secondary and primary education (reference category) as different levels. Health status was self-rated and included in our analysis with categories from excellent (1) as reference category, good (2), fair (3), poor (4) to very poor (5) [55].

Table 1 shows the proportion of women who visited breast or cervical screening in the period between 1992 and 1998 for every year. Table 2 displays the number (sum) of years in which a woman had attended for breast or cervical cancer screening during the 17 year period from 1992 to 2008. The uptake rate was 14.0 % for breast cancer screening and 22.0 % for cervical cancer screening over the whole analysed period. The average number of visits for breast or cervical cancer screening examinations over the whole period (1992 to 2008) was 2.38 and 3.78.

**Table 1:** Uptake rate for breast and cervical cancer screening during the 17 years period from 1992 to 2008

Health check-up	Breast cancer screening	Cervical cancer screening
1992	15.05%	29.62%
1993	14.69%	29.86%
1994	12.44%	26.78%
1995	14.10%	26.07%
1996	10.78%	25.95%
1997	13.74%	24.41%
1998	13.74%	24.76%
1999	12.56%	18.60%
2000	12.68%	21.68%
2001	14.22%	22.87%
2002	13.03%	18.48%
2003	15.28%	20.85%
2004	13.86%	17.54%
2005	14.34%	17.06%
2006	16.00%	16.82%
2007	15.88%	16.71%
2008	15.88%	15.17%
Total	14.02%	21.95%

Source: BHPS. Balanced panels consisted for breast cancer screening of 844 women from 11,816 observations

**Table 2:** Number of years with a breast or cervical screening visit during the 17 years period from 1992 to 2008

Health check-up	Breast cancer screening	Cervical cancer screening
0	39.6%	22.9%
1	11.7%	8.8%
2	9.7%	9.4%
3	8.5%	11.6%
4	10.7%	10.1%
5	3.8%	8.8%
6	6.3%	9.2%
7	3.3%	5.6%
8	2.8%	4.7%
9	1.8%	3.4%
10	0.8%	1.9%
11	0.2%	1.5%
12	0.2%	1.1%
13	0.1%	0.6%
14	0.4%	0.1%
15	0.0%	0.2%
16	0.0%	0.1%
17	0.0%	0.0%
Total	100.00%	100.00%

Source: BHPS. Balanced panels consisted for breast cancer screening of 844 women from 11,816 observations

The conditional probabilities of having a breast or cervical cancer screening in the recent year, dependent on having a breast or cervical cancer screening in the previous year are shown in Table 3. The conditional probabilities were especially higher for the same type of screening examination one and three years before.

**Table 3:** Conditional probabilities of a visit of a cancer screening visit in current year dependent from a visit in the previous year

Health check-up	Probability
Breast cancer screening (t) | Breast cancer screening (t-1)	0.334
Breast cancer screening (t) | Breast cancer screening (t-2)	0.302
Breast cancer screening (t) | Breast cancer screening (t-3)	0.462
Breast cancer screening (t) | Cervical cancer screening (t-1)	0.240
Breast cancer screening (t) | Cervical cancer screening (t-2)	0.233
Breast cancer screening (t) | Cervical cancer screening (t-3)	0.276
Cervical cancer screening (t) | Breast cancer screening (t-1)	0.140
Cervical cancer screening (t) | Breast cancer screening (t-2)	0.134
Cervical cancer screening (t) | Breast cancer screening (t-3)	0.151
Cervical cancer screening (t) | Cervical cancer screening (t-1)	0.393
Cervical cancer screening (t) | Cervical cancer screening (t-2)	0.320
Cervical cancer screening (t) | Cervical cancer screening (t-3)	0.488

Source: BHPS. Balanced panels consisted for breast cancer screening of 844 women from 11,816 observations

Table 4 presents descriptive statistics of the variables used in our estimates for the breast and cervical cancer screening examinations. The explanatory variables consist of individual and household characteristics. All time varying variables were averaged over the whole panel period and used in the auxiliary regressions for the Wooldridge-type estimators for determining their effect on the individual specific term.

**Table 4:** Descriptive characteristics for the balanced panels of breast and cervical cancer screening in Great Britain. Sample characteristics for the balanced sample of women from 1992 to 2008

Health check-up	Frequency or mean/SD
Total equivalised and deflated HH annual income (mean/SD)	3.10/(1.84)
Living with partner	0.72
Number of children in household (mean/SD)	0.52/(0.92)
Secondary education (ISCED)	0.42
Tertiary education (ISCED)	0.32
Employed part-time or full-time	0.52
GP visit during last 12 months	0.80
Health status good	0.47
Health status fair	0.23
Health status poor	0.07
Health status very poor	0.02
Status smoking	0.17
Moved residence within Great Britain	0.05
Region Scotland	0.08
Region Wales	0.05
Ethnic non-white	0.01
Age (mean/SD)	51.05/(15.57)

Source: BHPS. Balanced panels consisted for breast cancer screening of 844 women from 11,816 observations

The results for the dynamic univariate pooled probit model without random effects, initial conditions and the dynamic random effects panel probit model with initial conditions (Wooldridge estimator) for breast and cervical cancer screening are given in tables 5 and 6 3. The estimation of these and the following models use the same balanced sample of 844 individuals and 11816 observations. For both cancer screening examinations, taking part in screening examinations of the same type one year and three years before showed a strong positive influence on the current screening examination. These results were identical in both econometric specifications for breast and cervical cancer screening. For the univariate Wooldridge-type estimator, the coefficient was 0.108 for the first order own-effect lagged dependent variable and 0.790 for the third order own-effect lagged dependent variable. Similar results with a positive significant influence were found for the first order own-effect lagged dependent variable in the cervical cancer screening equation with a coefficient of 0.232 and a coefficient of 0.559 for the third order own-effect lagged dependent variable. Additionally, there were cross-lagged dependent variable (spillover) effects for the third order lag. The cross-lagged dependent variable effect for the third order lagged cervical cancer screening variable in the breast cancer screening regression was significant with a coefficient of 0.103. In a similar way the cross-lagged dependent variable effect for the third order lagged breast cancer screening examinations in the cervical cancer screening regression was significant with a coefficient of 0.187. It can be seen from Tables 5 and 6 that the coefficients for the dynamic univariate pooled probit and the dynamic random effects univariate panel probit model with initial conditions for breast and cervical in cancer screening were similar and the coefficients for the own-lagged dependent variables in the dynamic univariate pooled probit model were higher, overstating state dependence. A comparison of the results for the unbalanced and balanced panels for the dynamic pooled univariate probit and the univariate Wooldridge-type estimator showed similar results^4^.

**Table 5:** Estimates of the univariate pooled and dynamic RE panel probit breast cancer model

	Univariate pooled probit		Univariate RE panel probit	
	Coeff.	Robust SE	Coeff.	SE
Breast cancer screening one year before (t-1)	0.352***	(0.053)	0.108**	(0.049)
Breast cancer screening two years before (t-2)	0.205***	(0.046)	-0.018	(0.048)
Breast cancer screening three years before (t-3)	0.985***	(0.047)	0.790***	(0.044)
Cervical cancer screening one year before (t-1)	0.016	(0.041)	0.023	(0.046)
Cervical cancer screening two years before (t-2)	-0.056	(0.041)	-0.067	(0.046)
Cervical cancer screening three years before (t-3)	0.103***	(0.038)	0.103**	(0.044)
Breast cancer screening in 1992			0.092	(0.065)
Breast cancer screening in 1993			0.037	(0.065)
Breast cancer screening in 1994			0.173***	(0.067)
Cervical cancer screening in 1992			0.018	(0.051)
Cervical cancer screening in 1993			0.069	(0.052)
Cervical cancer screening in 1994			0.021	(0.055)
Averaged Total equivalised HH income/100			-0.015	(0.026)
Averaged Living with partner			0.111	(0.135)
Averaged Number of children in household			0.027	(0.065)
Averaged Secondary education (ISCED)			-0.586	(0.423)
Averaged Tertiary education (ISCED)			-0.483	(0.518)
Averaged employment status part-time or full-time			0.378***	(0.105)
Averaged GP visit during last 12 months			0.409***	(0.142)
Averaged Health status good			-0.018	(0.128)
Averaged Health status fair			-0.002	(0.151)
Averaged Health status poor			0.189	(0.248)
Averaged Health status very poor			-0.334	(0.441)
Averaged status smoker			0.100	(0.141)
Averaged Moved residence within GreatBritain			-0.413	(0.383)
Averaged age			0.030***	(0.004)
Total equivalised and deflated HH annual income	-0.009	(0.011)	0.004	(0.015)
Living with partner	0.087**	(0.044)	0.031	(0.116)
Number of children in household	-0.219***	(0.036)	-0.141***	(0.049)
Secondary education (ISCED)	-0.015	(0.043)	0.624	(0.419)
Tertiary education (ISCED)	0.011	(0.048)	0.561	(0.511)
Employed part-time or full-time	-0.128***	(0.041)	-0.212***	(0.065)
GP visit during last 12 months	0.250***	(0.048)	0.191***	(0.058)
Health status good	0.087*	(0.050)	0.066	(0.063)
Health status fair	0.058	(0.058)	0.011	(0.076)
Health status poor	0.060	(0.077)	0.002	(0.101)
Health status very poor	0.002	(0.133)	0.031	(0.152)
Status smoking	-0.133***	(0.051)	-0.207*	(0.124)
Moved residence within Great Britain	-0.029	(0.083)	0.046	(0.093)
Region Scotland	0.001	(0.070)	-0.001	(0.088)
Region Wales	0.074	(0.074)	0.156	(0.097)
Ethnic non-white	-0.001	(0.118)	0.074	(0.216)
Age between 50 and 64	0.628***	(0.043)	0.634***	(0.046)
Age between 65 and 70	-0.246***	(0.092)	-0.687***	(0.094)
Age 71 and older	-0.532***	(0.080)	-1.154***	(0.099)
Breast screening policy change	0.407***	(0.098)	0.556*	(0.105)
Constant	-1.681***	(0.086)	-3.746***	(0.277)
σ_α_			0.363***	(0.036)

Source: BHPS. Balanced panels consisted for breast cancer screening of 844 women from 11,816 observations. Robust SEs are displayed in parentheses, to account for individual repeated observations in the panel. *p<0.1; **p<0.05; ***p<0.01

**Table 6:** Estimates of the univariate pooled and dynamic RE panel probit cervical cancer model

	Univariate pooled probit		Univariate RE panel probit	
	Coeff.	Robust SE	Coeff.	SE
Breast cancer screening one year before (t-1)	0.046	(0.048)	0.010	(0.050)
Breast cancer screening two years before (t-2)	0.032	(0.048)	0.006	(0.051)
Breast cancer screening three years before (t-3)	0.205***	(0.047)	0.187***	(0.050)
Cervical cancer screening one year before (t-1)	0.423***	(0.038)	0.232***	(0.039)
Cervical cancer screening two years before (t-2)	-0.109***	(0.036)	-0.289***	(0.039)
Cervical cancer screening three years before (t-3)	0.729***	(0.039)	0.559***	(0.036)
Breast cancer screening in 1992			0.204***	(0.044)
Breast cancer screening in 1993			0.203***	(0.044)
Breast cancer screening in 1994			0.132***	(0.046)
Cervical cancer screening in 1992			-0.010	(0.072)
Cervical cancer screening in 1993			-0.055	(0.071)
Cervical cancer screening in 1994			0.239***	(0.069)
Averaged total equivalised HH income/100			0.015	(0.022)
Averaged living with partner			0.058	(0.107)
Averaged number of children in household			0.020	(0.046)
Averaged secondary education (ISCED)			-0.198	(0.383)
Averaged tertiary education (ISCED)			-0.296	(0.426)
Averaged employment status part-time or full-time			-0.051	(0.093)
Averaged GP visit during last 12 months			-0.068	(0.121)
Averaged health status good			-0.007	(0.107)
Averaged health status fair			-0.109	(0.136)
Averaged health status poor			0.189	(0.233)
Averaged health status very poor			0.148	(0.375)
Averaged status smoker			-0.135	(0.118)
Averaged moved residence within Great Britain			0.117	(0.286)
Averaged age			-0.007*	(0.004)
Total equivalised and deflated HH annual income	0.012	(0.009)	0.004	(0.013)
Living with partner	0.124***	(0.046)	0.099	(0.086)
Number of children in household	0.019	(0.017)	-0.001	(0.031)
Secondary education (ISCED)	0.015	(0.050)	0.196	(0.378)
Tertiary education (ISCED)	0.063	(0.053)	0.324	(0.418)
Employed part-time or full-time	0.039	(0.038)	0.060	(0.056)
GP visit during last 12 months	0.378***	(0.046)	0.422***	(0.048)
Health status good	-0.046	(0.039)	-0.042	(0.051)
Health status fair	-0.052	(0.046)	-0.037	(0.063)
Health status poor	-0.035	(0.064)	-0.067	(0.089)
Health status very poor	-0.140	(0.106)	-0.258*	(0.145)
Status smoking	-0.025	(0.063)	-0.056	(0.070)
Moved residence within Great Britain	0.037	(0.045)	0.137	(0.100)
Region Scotland	0.048	(0.051)	0.051	(0.074)
Region Wales	-0.109	(0.081)	-0.111	(0.092)
Ethnic non-white	-0.200*	(0.109)	-0.272	(0.187)
Age between 20 and 24	0.596***	(0.143)	0.543***	(0.175)
Age between 25 and 49	0.453***	(0.065)	0.414***	(0.092)
Age between 50 and 64	0.191***	(0.055)	0.208***	(0.068)
Age 65 and older	-0.747***	(0.069)	-0.763***	(0.085)
Cervical screening policy change	-0.072*	(0.041)	-0.114*	(0.045)
Constant	-1.818***	(0.087)	-1.532***	(0.266)
σ_α_			0.349***	(0.031)

Source: BHPS. Balanced panels consisted for cervical cancer screening of 844 women from 11,816 observations. Robust SEs are displayed in parentheses, to account for individual repeated observations in the panel. *p<0.1; **p<0.05; ***p<0.01

To obtain valid estimates for the univariate estimation of breast and cervical cancer screening in comparison to the bivariate estimation, the implicit assumptions were made that the correlation of the individual specific random effects terms across the 2 equations was equal to 0 and the coefficients of the cross-lagged variables for the screening examinations were equal to 0. Shown in Table 7 are the estimations for the uptake of breast and cervical cancer screening in Great Britain with a dynamic bivariate pooled probit model and the dynamic random effects bivariate panel probit with initial conditions (bivariate Wooldridge-type) estimator. The own- and cross-lagged dependent variable effects were also visible in the dynamic bivariate pooled probit model and in the dynamic random effects bivariate panel probit model estimations. In the breast cancer screening equation, the coefficient for the first order own-effect lagged dependent variable was 0.116; the coefficient for the third order own-effect lagged dependent variable was 0.797 for the bivariate Wooldridge-type estimators (specification with a GP visit during the last 12 months). Similar results exist in the cervical cancer screening equation for the first order own-effect lagged dependent variable with a coefficient of 0.227 and a coefficient of 0.555 for the third order own-effect lagged dependent variable. For the third order cross-lagged dependent variable, the cervical coefficient was 0.083 in the breast cancer screening equation and the breast coefficient in the cervical cancer screening equation was 0.181. The dynamic bivariate pooled probit model and the dynamic random effects bivariate panel probit showed similar coefficients for the socioeconomic variables and the coefficients for the lagged dependent variables were higher in the first model, because of the exogeneity assumption.

**Table 7:** Estimates of the bivariate pooled and dynamic RE panel probit breast and cervical cancer model

	Bivariate pooled probit		Bivariate pooled probit		Bivariate RE panel probit		Bivariate RE panel probit		Bivariate RE panel probit without GP			
	Breast		Cervical		Breast		Cervical		Breast		Cervical	
	Coeff.	Robust SE	Coeff.	SE	Coeff.	SE	Coeff.	SE	Coeff.	SE	Coeff.	SE
Breast cancer screening 1 year before	0.351***	(0.053)	0.052	(0.048)	0.116**	(0.048)	-0.005	(0.052)	0.116**	(0.048)	0.001	(0.052)
Breast cancer screening 2 years before	0.207***	(0.046)	0.043	(0.048)	-0.009	(0.048)	-0.002	(0.052)	-0.009	(0.048)	0.005	(0.052)
Breast cancer screening 3 years before	0.986***	(0.047)	0.216***	(0.047)	0.797***	(0.044)	0.181***	(0.052)	0.795***	(0.044)	0.187***	(0.052)
Cervical cancer screening 1 year before	0.009	(0.041)	0.419***	(0.038)	-0.005	(0.049)	0.227***	(0.039)	0.005	(0.049)	0.237***	(0.039)
Cervical cancer screening 2 years before	-0.060	(0.041)	-0.110***	(0.036)	-0.087*	(0.048)	-0.291***	(0.039)	0.083*	(0.048)	-0.279***	(0.039)
Cervical cancer screening 3 years before	0.100***	(0.038)	0.726***	(0.039)	0.083*	(0.046)	0.555***	(0.036)	0.089*	(0.046)	0.559***	(0.036)
Breast cancer screening in 1992					0.096	(0.064)	0.001	(0.072)	0.103	(0.065)	-0.005	(0.071)
Breast cancer screening in 1993					0.034	(0.064)	-0.045	(0.072)	0.061	(0.065)	-0.035	(0.071)
Breast cancer screening in 1994					0.173***	(0.066)	0.258***	(0.070)	0.204***	(0.067)	0.265***	(0.069)
Cervical cancer screening in 1992					0.011	(0.051)	0.204***	(0.044)	0.017	(0.051)	0.202***	(0.044)
Cervical cancer screening in 1993					0.068	(0.051)	0.206***	(0.045)	0.076	(0.052)	0.204***	(0.044)
Cervical cancer screening in 1994					0.034	(0.055)	0.134***	(0.046)	0.057	(0.055)	0.142***	(0.046)
Longitudinal averaged X_it_					Yes		Yes		Yes		Yes	
Total equivalised HH annual income	-0.009	(0.011)	0.012	(0.009)	0.004	(0.015)	0.004	(0.013)	0.004	(0.015)	0.002	(0.013)
Living with partner	0.088**	(0.044)	0.122***	(0.046)	0.036	(0.115)	0.098	(0.086)	0.034	(0.115)	0.095	(0.085)
Number of children in household	-0.218***	(0.036)	0.012	(0.017)	-0.139***	(0.049)	-0.003	(0.031)	-0.142***	(0.049)	-0.012	(0.031)
Secondary education (ISCED)	-0.014	(0.043)	0.012	(0.050)	0.676	(0.423)	0.213	(0.380)	0.662	(0.424)	0.188	(0.378)
Tertiary education (ISCED)	0.012	(0.048)	0.059	(0.053)	0.618	(0.514)	0.349	(0.421)	0.574	(0.514)	0.310	(0.418)
Employed part-time or full-time	-0.126***	(0.041)	0.026	(0.038)	-0.205***	(0.065)	0.052	(0.056)	-0.210***	(0.065)	0.043	(0.056)
GP visit during last 12 months	0.254***	(0.048)	0.377***	(0.046)	0.195***	(0.058)	0.422***	(0.048)	-	-	-	-
Health status good	0.085*	(0.050)	-0.045	(0.039)	0.068	(0.063)	-0.041	(0.051)	0.093	0.062	0.018	(0.049)
Health status fair	0.056	(0.058)	-0.052	(0.046)	0.012	(0.076)	-0.037	(0.063)	0.047	(0.075)	0.049	(0.061)
Health status poor	0.055	(0.078)	-0.037	(0.064)	0.002	(0.101)	-0.068	(0.089)	0.042	(0.100)	0.033	(0.088)
Health status very poor	0.007	(0.133)	-0.146	(0.107)	0.045	(0.151)	-0.261*	(0.145)	0.089	(0.151)	-0.161	(0.144)
Status smoking	-0.129**	(0.051)	-0.031	(0.063)	-0.207*	(0.123)	0.131	(0.100)	-0.221*	(0.123)	0.095	(0.100)
Moved residence within Great Britain	-0.028	(0.082)	0.033	(0.045)	0.051	(0.093)	-0.060	(0.070)	0.052	(0.093)	-0.065	(0.069)
Region Scotland	0.005	(0.069)	0.049	(0.051)	0.013	(0.086)	0.059	(0.074)	0.032	(0.087)	0.080	(0.073)
Region Wales	0.071	(0.074)	-0.112	(0.081)	0.144	(0.096)	-0.112	(0.091)	0.155	(0.097)	-0.104	(0.090)
Ethnic non-white	0.001	(0.120)	-0.201*	(0.107)	0.073	(0.211)	-0.269	(0.188)	0.072	(0.217)	-0.255	(0.186)
Age between 50 and 64	0.625***	(0.043)			0.630***	(0.046)			0.628***	(0.046)		
Age between 65 and 70	-0.269***	(0.092)			-0.706***	(0.094)			-0.690***	(0.095)		
Age 71 and older	-0.529***	(0.080)			-1.144***	(0.098)			-1.114***	(0.099)		
Age between 20 and 24			0.678***	(0.143)			0.621***	(0.176)			0.644***	(0.175)
Age between 25 and 49			0.528***	(0.066)			0.480***	(0.093)			0.481***	(0.093)
Age between 50 and 64			0.238***	(0.055)			0.248***	(0.068)			0.242***	(0.046)
Age 65 and older			-0.707***	(0.069)			-0.751***	(0.085)			-0.722***	(0.084)
Breast screening policy change	0.426***	(0.099)			0.574***	(0.104)			-0.722***	(0.084)		
Cervical screening policy change			-0.076*	(0.041)			-0.118***	(0.045)			-0.140***	(0.044)
Constant	-1.680***	(0.086)	-1.856***	(0.087)	-3.676***	(0.270)	-1.633	(0.268)			-1.432***	(0.258)
σ_α1_					0.355***		(0.036)		0.370***		(0.035)	
σ_α2_					0.359***		(0.032)		0.352***		(0.031)	
ρ	0.173***		(0.028)		0.170***		(0.026)		0.179***		(0.298)	
ρ_α_					0.219*		(0.121)		0.214*		(0.117)	

Source: BHPS. Balanced panels consisted for breast and cervical cancer screening of 844 women from 11,816 observations. Robust SEs are displayed in parentheses, to account for individual repeated observations in the panel. *p<0.1; **p<0.05; ***p<0.01

We investigated whether the two equations could be estimated separately, because the nonzero coefficients of the cross-lagged dependent variables imply that the equations can only be separated if the correlation of the individual specific random effects and the correlation of the error terms are zero. The results of the bivariate Wooldridge-type estimator show that first the hypothesis of independence between the error terms with a correlation had to be rejected, because *ρ* with a value of 0.170 was significantly different from 0. Also the individual specific random effects terms were correlated, because *ρ*_*a*_ with a value of 0.219 was statistically significant different from 0.

Higher uptake for breast and cervical cancer screening examinations was observed for women who visited their GP within the previous year. Women aged 50 to 64 had an increased uptake for breast cancer screening examinations in comparison with women age 16 to 49 (reference category); women age 25 to 49 had an increased uptake for cervical cancer screening examinations in comparison with the reference category of women age 16 to 19. Women age 65 to 70 had an increased uptake of breast cancer screening after the change of policy rules in 2002. However, for women age 25 to 49, the uptake did not increase for cervical cancer screening after the change of policy rules for recall in 2003. For the breast cancer screening equation, the variables that had a negative impact were having a higher number of children and being employed, but these variables did not have a negative impact on the cervical cancer screening equation. Both cancer screening equations were not influenced by the levels of self-assessed health status, smoking status, household income or living with a partner. A further specification for testing the possible endogeneity of a GP visit in the dynamic random effects bivariate panel probit model by not including this variable gave very similar results in comparison to the specification with a GP visit.

## Discussion

Our analysis of the BHPS investigated for the first time the determinants of screening uptake for breast and cervical cancer screening and possible spillover effects with a dynamic bivariate panel probit model. A dynamic random effects bivariate panel probit model was used for the estimation over a period for 17 years from 1992 to 2008 for Great Britain with a balanced panel. The uptake of breast and cervical cancer screening was modelled with lagged dependent variables up to order 3 and it was controlled for individual heterogeneity. The strong influence of past screening behaviour with a highly significant effect of the own first order lag and third order lag for breast and cervical cancer screening shows that past screening behaviour influences actual behaviour. These results can be interpreted as persistence in screening behaviour and state dependence [56]. The reasons for the strong positive state dependence with respect to the effects of own lagged dependent variables for breast and cervical cancer screening are the adherence to screening policy rules for the invitation in Great Britain. The NHS Breast and Cervical Screening Programmes give explicit rules for the time interval between screening examinations [7, 11]. The importance of these screening rules on current behaviour can especially be seen in the high predictive value of the same type of screening examination three years before. The coefficients for the same type of screening examinations one year before were significantly positive for both types of cancer screening examinations. Necessary control screening examinations checked unclear test results from the previous health check-up one year year before and could especially explain the coefficient for the first order lag. However, with existing data from the BHPS, it is not possible to differentiate between these different possibilities. Initial conditions show relevance for both types of screening examinations. If initial conditions for the first three years had not been taken into account, the influence of past screening behaviour on actual behaviour would have been overestimated.

Our empirical investigation showed additionally the importance of cross-lagged dependent variable effects for the third order lags for both types of screening examinations. Visiting one type of screening examination enhanced for the third order lagged variable of one type of screening examination the uptake of the other screening examination. This result could be explained by a spillover effect that an individual woman is more accessible for preventive information after visiting one type of screening examination in the past. Another possibility could be that unobserved variables are correlated with the lagged dependent variables. Heterogeneity with unobserved characteristics plays a role for breast and cervical cancer screening. It is responsible for about one third of the unsystematic variation in each of the equations. The correlation of the two individual specific random terms is positive and significant at a 10-% level. This means that the persistent unobserved characteristics of a woman, which are also responsible for a higher or lower uptake of breast cancer screening examination can explain the higher or lower uptake of a cervical cancer screening examination and vice versa. The significant positive correlation of the error terms means has as a consequence that idiosyncratic events or shocks in a time period that influences the decision of a woman to visit one type of cancer screening examination also influences the decision of a woman to visit the other type of cancer screening examination. The significance of the cross-lagged variables and the significant correlation of individual specific random terms and error terms show that both types of screening examinations are simultaneously determined and interrelated and that both equations cannot be estimated separately and have to be estimated jointly [49]. Therefore, screening examinations cannot be analysed independent from each other and a change in the screening policy rules of one examination would influence its own uptake, but it would also influence the uptake of the other screening examination, because of possible spillover effects and a decision maker should be aware of these effects.

Considering the effect of the other covariates for breast and cervical cancer screening the uptake is not explained by all the same variables in both screening examinations. The variables age and a previous GP visit have the same influence for both screening examinations. The relevance of the policy rules for the invitation with specified age intervals for cervical and breast cancer screening within both programmes can be seen in our specifications with the highest probability of uptake in the recommended age groups and this effect can also be seen in other analyses [6, 30]. The finding of a lower screening uptake in the oldest age group in comparison to younger age groups is in accordance with the shorter pay-off period for older women from the human capital theory approach. Furthermore, a GP visit in the last year leads to a higher uptake of breast and cervical cancer screening. The GP plays an important role as gatekeeper and in prevention by referring a patient for specific screening examinations (e.g. breast and cervical cancer screening) or by doing the screening examination as is in the case for cervical cancer screening [37]. Our results are similar to those of an Italian study which analysed the uptake of breast and cervical cancer screening with a recursive probit. Estimations from the Italian study showed that GP visits led to an increased uptake of breast and cervical screening [29].

Health status can be interpreted as a proxy for health and women with a poor self-assessed health status could have a high demand for these two cancer screening examinations in order to invest in prevention activities and to increase their health stock. However, poor self-assessed health status can influence uptake also in other ways such as changed perceptions on the preventability of health problems and diseases. Individuals with a poorer health status also expressed less interest in receiving prevention information [57]. Psychological factors, such as fear and anxiety about confirmation of a disease, can be related to a poor health status and this correlation could be especially relevant for the both analysed female cancer screening examinations. Also, women with a poor health status may be unable to visit the screening location. These possibilities of influence could cancel one another out. Individual and household characteristics such as employment, number of children in the household and smoking had a non-uniform influence on both uptakes. In a systematic review which analysed the determinants of screening uptake for different cancer screenings, none of the analysed socioeconomic variables had the same influence in all cancer screening examinations [27]. There are similar results for some socioeconomic variables and health-related variables, however there are also some differences when our results on the uptake of breast and cervical cancer screening are compared with other studies which had analysed the uptake behaviour for Great Britain and used the BHPS. Analysis of breast cancer screening uptake with the BHPS was done in one study with a balanced sample [6]. Identical results were found for the relevance of previous screening history, a GP visit, age and self-assessed health status. However, results were different to own results for education level, marital status and the averaged income term (Mundlak term), because they were significant in this analysis. Analysis of cervical cancer screening uptake with the BHPS was done in a further analysis with a balanced sample [5]. In this analysis, previous screening history, age and a GP visit were significant for cervical cancer screening in Great Britain. Our results were confirmed by another study which analysed the uptake of cervical cancer screening uptake in England with an unbalanced panel for the first 12 waves of the BHPS until 2003 [30]. Only one analysis compared the sociodemographic determinants for the uptake of breast and cervical cancer screening at the same time for Great Britain with a cross-sectional survey [44]. Results for the effects of determinants on the uptake of both female cancer screening examinations were different, because level of education, occupational classification and ethnicity were not significant; only indicators for wealth were positively significant. For having a smear test, a higher educational level, and white British ethnicity were positively significant. But indicators for wealth or occupational classification were not significant. This is one of the few studies that compared the determinants of the uptake of breast and cervical screening to find different determinants to be responsible for the uptake of both screening examinations. An advantage of this analysis lies in the fact that the same estimation sample was used for both screening examinations. However, unobserved heterogeneity and state dependency could not be taken into account with cross-sectional data in this analysis and this could explain the different results to the results of our own study.

Several limitations exist in our analysis. First, there was no information about results from previous mammographies and smear tests available and so it cannot be decided if the screening test is a reexamination screening examination according to the policy rules or a control examination because of inconclusive test results one year before. Second, visiting breast and cervical cancer screening examinations is self-reported and could be influenced by a recall bias [58]. Third, no personal or family history of breast or cervical cancer was available in the BHPS and women with a family history of cancer belong to a high risk population. For these women, more frequent mammography was not recommended until recently. It is now recommended by the National Institute for Health and Care Excellence (NICE) that women with a family history of breast cancer should start having screening mammograms every year in their 40’s [59]. It has been shown that these women can have a higher uptake of both screening examinations [60, 61]. Fourth, there was no information about the level of trust in the GP or in the NHS available. It has been shown that doing a breast cancer or cervical cancer screening can be dependent on the trust in these institutions [29]. Fifth, no information about the characteristics of the screening unit was available. Characteristics of the screening unit such as structure and organization of medical services performing the screening test can influence the uptake rate. This association has been shown for cervical cancer screening uptake and general practice factors in England [62]. A sixth limitation comes from not using detailed microgeographic information, because uptake rates for health check-ups can be higher in affluent and less deprived areas [63]. Seventh, there was no information about women’s attitude to being screened and belief about the attitudes of near relatives although it has been shown that these attitudes can also be of significance for the uptake rate [64].

## Conclusions

The innovative feature of our article is the simultaneous analysis of uptake of breast and cervical cancer screening using a dynamic random effects bivariate panel probit model with initial conditions (Wooldridge-type estimator for a bivariate panel probit) for a balanced sample. No research exists until now on how different types of screening examinations can influence each other. Our investigation shows the high relevance of past screening behaviour and state dependency for the same type of cancer screening. Past uptake of the same type of screening examination increases the chances of recent uptake for a breast or cervical cancer screening examination even after controlling for covariates and unobserved heterogeneity. Additionally, there were dynamic spillover effects from one type of cancer screening examination to the other type of cancer screening examination for the third order lag. Error terms of both equations and individual specific terms are contemporaneously correlated which as a consequence means that the uptake of both screening examinations has to be estimated simultaneously. A policy implication of our results is that promoting uptake of one type of prevention activity can enhance the uptake of the other type of prevention activity.

## Abbreviations

BHPS: British Household Panel Survey
GP: General practitioner
GUM: Genito-urinary medicine
LBC: Liquid based cytology
NHS: National Health Service
NHSBSP: NHS Breast Screening Programme
NHSCSP: NHS Cervical Screening Programme
NICE: National Institute for Health and Care Excellence
RE: Random effects
UK: United Kingdom

## Footnotes

1 As later explained in the methods section Northern Ireland was excluded from our analysis with the used dataset (BHPS) and so the term Great Britain is used instead of United Kingdom (UK).

2 The Wooldridge estimator for the bivariate case is estimated as a simulated Maximum Likelihood estimator with 2RN Halton draws. N is the number of individuals and R is the number of replications and we used for the number of replications 60.

3 The coefficients of the dynamic random effects panel probit and the pooled panel probit model have different normalizations. For comparing the coefficients of both estimations it is necessary to rescale the coefficients of one equation, i.e. the coefficients of the dynamic random effects model have to be multiplied by ${\left(1-{\sigma}_{\alpha}^2\right)}^{-1/2}$ if they are compared with dynamic pooled probit model.

4 Results for the unbalanced panel estimations are available from the authors on request.
